# The dynamic changes and influencing factors of visual symptoms after small incision lenticule extraction

**DOI:** 10.1186/s12886-023-02964-8

**Published:** 2023-05-19

**Authors:** Han Chen, Xiuyu Mao, Dongye Xu, Chenwen Guo, Jinhui Dai

**Affiliations:** 1 Department of Ophthalmology, Fujian Provincial Hospital, Fuzhou, Fujian China; 2 Department of Ophthalmology, Zhongshan Hospital of Fudan University, No.180 Fenglin Road, Shanghai, 200032 China; 3 Shengli Clinical Medical College of Fujian Medical University, Fuzhou, Fujian China; 4 Department of Ophthalmology, Eye and ENT Hospital of Fudan University, Shanghai, China; 5 Department of Ophthalmology, Zhongshan Hospital of Fudan University (Xiamen Branch), Xiamen, Fujian China

**Keywords:** Small incision lenticule extraction (SMILE), Subjective visual quality, Visual symptoms, Patient-reported outcome, Higher order aberrations

## Abstract

**Background:**

To investigate the dynamic changes and influencing factors of visual symptoms after small incision lenticule extraction (SMILE).

**Methods:**

This was a prospective observational study. Visual symptoms including glare, haloes, starbursts, hazy vision, fluctuation, blurred vision, double vision and focusing difficulties were evaluated before and 1, 3, 6 months after SMILE using a questionnaire. Generalized linear mixed models were used to assess the effects of preoperative characteristics and objective visual quality parameters on postoperative visual symptoms.

**Results:**

73 patients/146 eyes were enrolled. Preoperatively, the most common symptoms were glare (55% of eyes), haloes (48%), starbursts (44%) and blurred vision (37%). At 1 month postoperatively, the incidence and extent scores of glare, haloes, hazy vision and fluctuation rose significantly. At 3 months, the incidence and extent scores of glare, haloes and hazy vision restored to baseline. And at 6 months, the extent scores of fluctuation returned to baseline. Other symptoms (e.g., starbursts) did not change before and 1, 3, 6 months after SMILE. Preoperative visual symptoms were associated with postoperative symptoms, as patients with a symptom preoperatively had higher postoperative scores for that symptom. Age was related to postoperative extent of double vision (coefficient = 0.12, *P* = 0.046). There were no significant associations between postoperative visual symptoms and preoperative SE, scotopic pupil size, angle kappa (with intraoperative adjustment), postoperative HOAs or scattering indexes.

**Conclusions:**

The incidence and extent scores of hazy vision, glare, haloes and fluctuation increased at the first month after SMILE, and recovered to baseline at 3 or 6 months. Preoperative visual symptoms were associated with the postoperative symptoms and should be fully considered before SMILE.

**Supplementary Information:**

The online version contains supplementary material available at 10.1186/s12886-023-02964-8.

## Background

Small incision lenticule extraction (SMILE) is one of the most widely performed refractive surgery worldwide, which is minimally invasive, safe, effective and stable [1–4]. However, some patients still reported visual symptoms such as glare postoperatively, though their uncorrected distant visual acuity (UDVA) reached 20/20 [5].

Some scholars have investigated the patient-reported outcome of visual quality after SMILE, reporting that 50-70% of patients experienced postoperative glare, vision fluctuation or haloes [5–8]. However, the lack of preoperative data on visual symptoms in these studies might lead to an overestimation and exaggeration of the actual SMILE-induced visual symptoms. For an accurate assessment of the subjective visual quality after SMILE, a sufficient evaluation of preoperative visual complaints was necessary.

To date, little is known about how visual symptoms dynamically change from preoperative to postoperative period of SMILE. Furthermore, no previous studies have investigated the impact of the preoperative visual symptoms on post-SMILE visual symptoms.

Also, controversy exists about other potential influencing factors for postoperative visual symptoms. Surgery-induced higher order aberrations (HOAs) were speculated to play roles in vision complaints after laser in situ keratomileusis (LASIK) [9, 10], but not SMILE [7, 8, 11–13]. Age, preoperative refraction and pupil size were observed to be related to post-SMILE aberrations [5, 11, 14], but it is unclear if they are also linked to visual symptoms.

This study demonstrated the dynamic changes in visual symptoms from pre-SMILE to 6-month post-SMILE, and revealed the associations between preoperative visual symptoms and postoperative symptoms. In addition, the effects of age, scotopic pupil size, preoperative spherical equivalent (SE), angle kappa, postoperative HOAs and scattering indexes on postoperative visual complaints were also analyzed.

## Methods

### Study population

This prospective study enrolled 73 myopic and astigmatism patients (146 eyes) who underwent SMILE in Eye and ENT Hospital of Fudan University from July 2020 to November 2020. Inclusion criteria were as follows: (1) age range between 18 and 45 years; (2) spherical diopters of -0.5 D ~ -10 D, and astigmatism diopters no greater than 5 D; (3) corrected distant visual acuity (CDVA) ≥ 20/25; (4) stable refractive status for at least 2 years; (5) soft contact lenses discontinued for at least 1 week and rigid gas permeable contact lens for at least 3 weeks. Exclusion criteria were as follows: (1) central corneal thickness (CCT) less than 480 μm; (2) estimated postoperative residual stromal bed thickness less than 280 μm; (3) patients with keratoconus, corneal scars, severe dry eye, glaucoma, retina detachment or other intraocular diseases; (4) patients with history of ophthalmic surgery or trauma; (5) patients with connective tissue diseases or other systemic chronic diseases.

Questionnaire on visual symptoms and comprehensive ophthalmic examinations were conducted preoperatively and 1, 3, 6 months postoperatively. Examinations consisted of slit-lamp examination, measurements of UDVA, corrected distant visual acuity (CDVA), manifest refraction, corneal topography (Pentacam HR, Oculus), scotopic pupil size, ocular HOAs and scattering indexes.

### Questionnaire on visual symptoms

This questionnaire was designed to collect the information on patients’ visual symptoms. Patients completed the questionnaire at preoperative and postoperative 1, 3 and 6 months. The translated version of questionnaire is presented in the appendix. The questionnaire consisted of eight visual symptoms, including glare, haloes, starbursts, hazy vision, blurred vision, double vision, fluctuation in vision and focusing difficulties. We explained the specific meaning of each visual symptom to the patients. For the first six symptoms, example pictures were attached for illustration. Patients were requested to grade the extent of visual symptoms for each eye (no symptom [0], mild [1], moderate [2], severe [3]). Before answering the questionnaire, patients were given sufficient time to recall the presence and severity of any visual symptoms they experienced in their daily life, and they were allowed to compare their eyes in a dimly lit examination room. The visual symptoms under correction with spectacles or contact lenses were examined before SMILE.

### Scotopic pupil size and angle kappa

The scotopic pupil size, estimated from horizontal pupil diameter, was measured with ARK-1 auto refractometer (Nidek) in a dark room after an adaptation for 10 min.

Angle kappa, referring to the angle between visual axis and pupillary axis, was estimated using Pentacam HR in this study. The angle kappa measured by Pentacam HR was actually the distance between pupil center and corneal apex, reported in a polar coordinate as a chord distance (mm).

### Ocular HOAs and scattering indexes

Ocular HOAs were evaluated with OPD Scan III (Nidek). The pupil diameter was set to 5 mm. The root mean square (RMS) values of following ocular aberrations were documented, including coma, spherical aberration (SA), trefoil and total HOAs (order S3 to order S6).

Visual quality parameters including objective scattering indexes (OSI), modulation transfer function cut-off frequency (MTF_cutoff_) and Strehl ratio (SR) was measured by OQAS II (Visiometrics S.L.), which was based on the double-pass technique. OSI was referred to the ratio of light intensity between the peripheral retina image (12’ ~ 20’) and the central region (1’). MTF_cutoff_ was the spatial frequency corresponding to the MTF value of 0.01. And SR was the ratio of the area under the MTF curve of the eye with aberration to the aberration-free eye.

### Surgery procedure

SMILE surgery was performed by the same experienced surgeon (JD), using the VisuMax 500-kHz Femtosecond Laser System (Carl Zeiss Meditec AG). The pulse energy was set to 130 nJ, cap thickness 120 μm, cap diameter 7.6 mm, side-cut angle 90°, and 2-mm incision at 10:30 o’clock position. Postoperative treatment regimen included 0.5% levofloxacin eye drops 4 times daily for a week, 0.1% sodium hyaluronate eye drops 4 times daily for 3 months, and 0.1% fluorometholone eye drops 6 times daily and tapered within one month.

Of note, based on Pentacam HR scans and Hirschberg corneal reflex tests, the magnitude and direction of apparent angle kappa were recorded preoperatively, in order to make the center of suction ring closer to the visual axis during operation.

### Statistical analysis

The binocular data of each patient were included for analysis. Generalized estimating equation (GEE) with Sidak post hoc test was used to adjust the correlation between eyes from the same patient, and to compare the incidence and extent scores of visual symptoms, and repeatedly measured ophthalmic parameters at different time points. A generalized linear mixed model (GLMM) with multinomial distribution and logit link function was used to identify influencing factors for postoperative visual symptom scores. Each participant and every follow-up visit were treated as random effects in the GLMM analysis with eyes nested within participants. The GLMM analysis incorporated various factors, including preoperative visual symptoms, age, scotopic pupil size, preoperative SE, angle kappa, postoperative ocular HOAs (coma, SA and trefoil), OSI, MTF_cutoff_ and SR. All data were analyzed using SPSS for macOS (v. 26.0, IBM). Statistical significance was defined as *P* value (two sided) less than 0.05.

## Results

**Fig. 1:**
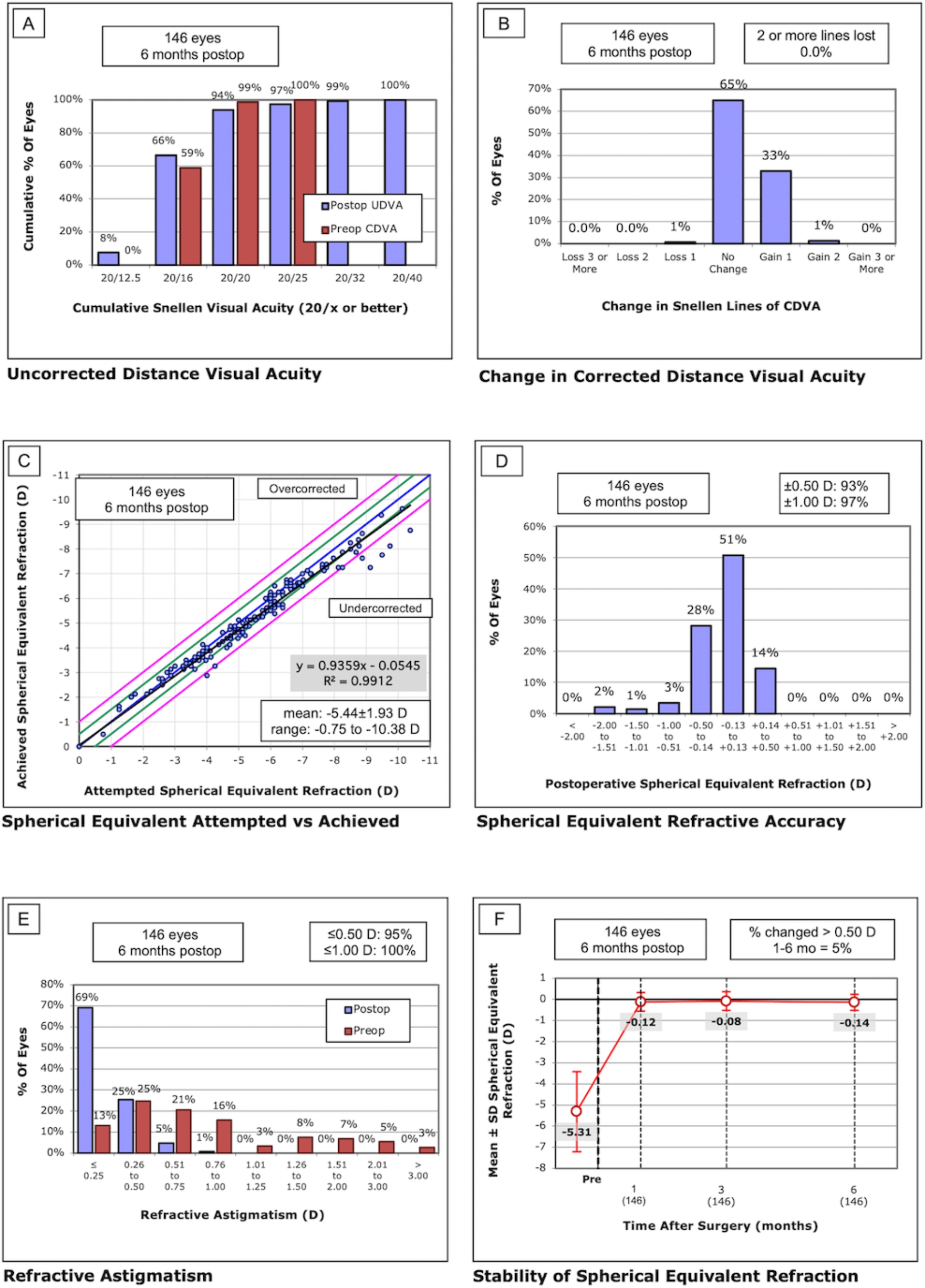
Refractive outcomes after SMILE. A: cumulative corrected distant visual acuity (CDVA) before SMILE versus cumulative uncorrected distant visual acuity (UDVA) at 6 months after SMILE; B: changes in Snellen lines of CDVA; C: attempted spherical equivalent (SE) versus achieved SE at 6 months after SMILE; D: SE refraction at 6 months after SMILE; E: refractive astigmatism before SMILE versus 6 months after SMILE; F: the mean SE at different time points after surgery.

146 eyes from 73 patients (21 males [29%], 52 females [71%]) were enrolled. Table 1 presented the demographic characteristics. The refractive outcomes of SMILE, including safety, efficacy and predictability were depicted in Fig. 1 and Supplemental Table 1. There were no significant differences in refractive outcomes between postoperative 6 months and 1 month.

**Table 1:** Demographic characteristics

Characteristics	Mean ± SD	Range
Age (years)	25.63 ± 6.41	18, 44
CDVA (logMAR)	-0.03 ± 0.03	-0.08, 0.05
Spherical (D)	-4.82 ± 1.86	-9.25, -0.50
Cylinder (D)	-0.98 ± 0.73	-3.75, 0
SE (D)	-5.31 ± 1.89	-10.13, -0.75
AL (mm)	25.78 ± 1.14	23.09, 29.98
IOP (mmHg)	15.49 ± 2.89	9.8, 21.2
CCT (µm)	545.44 ± 26.49	480, 607
Scotopic pupil size (mm)	6.83 ± 0.71	4.7, 8.2

AL = ocular axil length; CCT = central corneal thickness; CDVA = corrected distant visual acuity; IOP = intraocular pressure; SE = spherical equivalent

### Ocular HOAs and scattering indexes

**Fig. 2:**
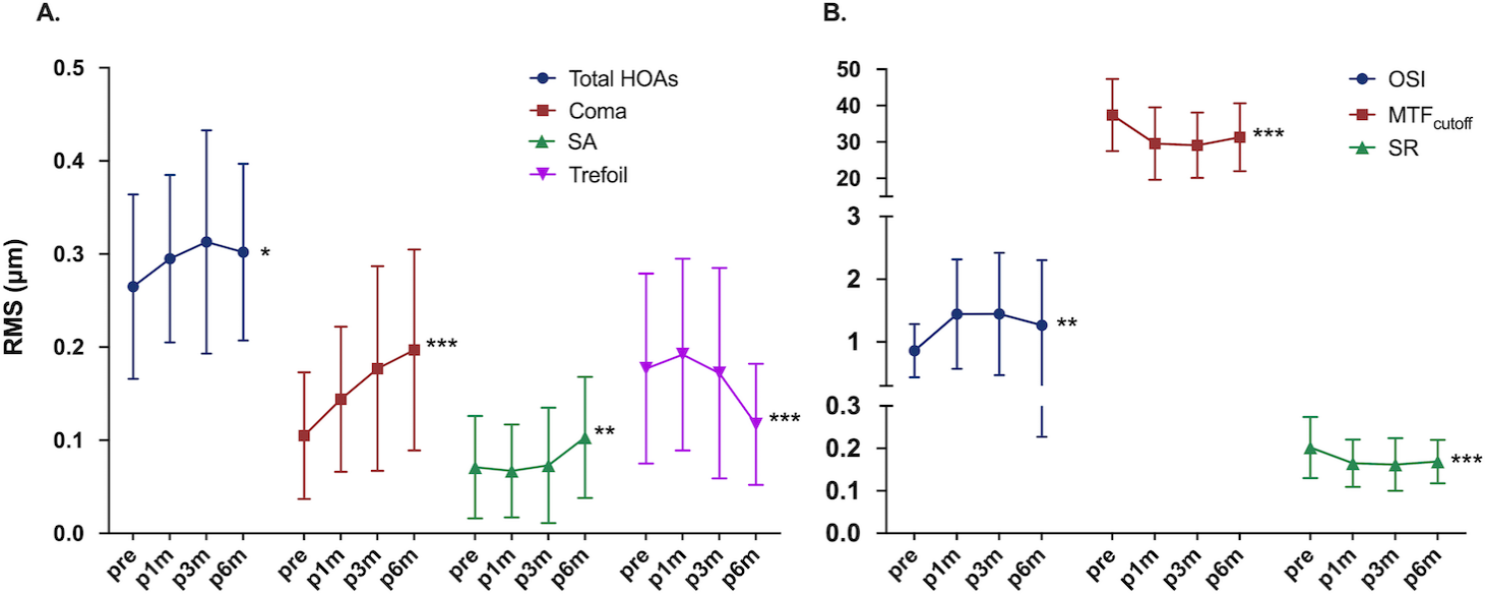
Ocular HOAs and scattering indexes before and after SMILE. The generalized estimating equation (GEE) with Sidak post hoc test was applied for comparing objective visual parameters between different time points; *, ** and *** indicated *P* < 0.05, *P* < 0.01 and *P* < 0.001, respectively (6 months postoperatively versus preoperatively). MTF_cutoff_**=** modulation transfer function cut-off frequency; OSI = objective scattering index; SA = spherical aberration; SR = Strehl ratio; total HOAs = order S3 to order S6 ocular aberrations

Figure 2 summarized the ocular HOAs and scattering indexes before and after SMILE. At 6 months after SMILE, the ocular total HOAs, coma and SA were significantly higher than preoperative level (*P* = 0.02, *P* < 0.001 and *P* = 0.001 respectively). The ocular trefoil increased slightly at 1 month, and experienced a decrease at 6 months (*P* < 0.001). The OSI, MTF_cutoff_ and SR all changed dramatically at 1 month after SMILE, and the changes were still significant at 6 months (*P* = 0.004, *P* < 0.001 and *P* < 0.001 respectively).

### Dynamic changes in visual symptoms

**Fig. 3:**
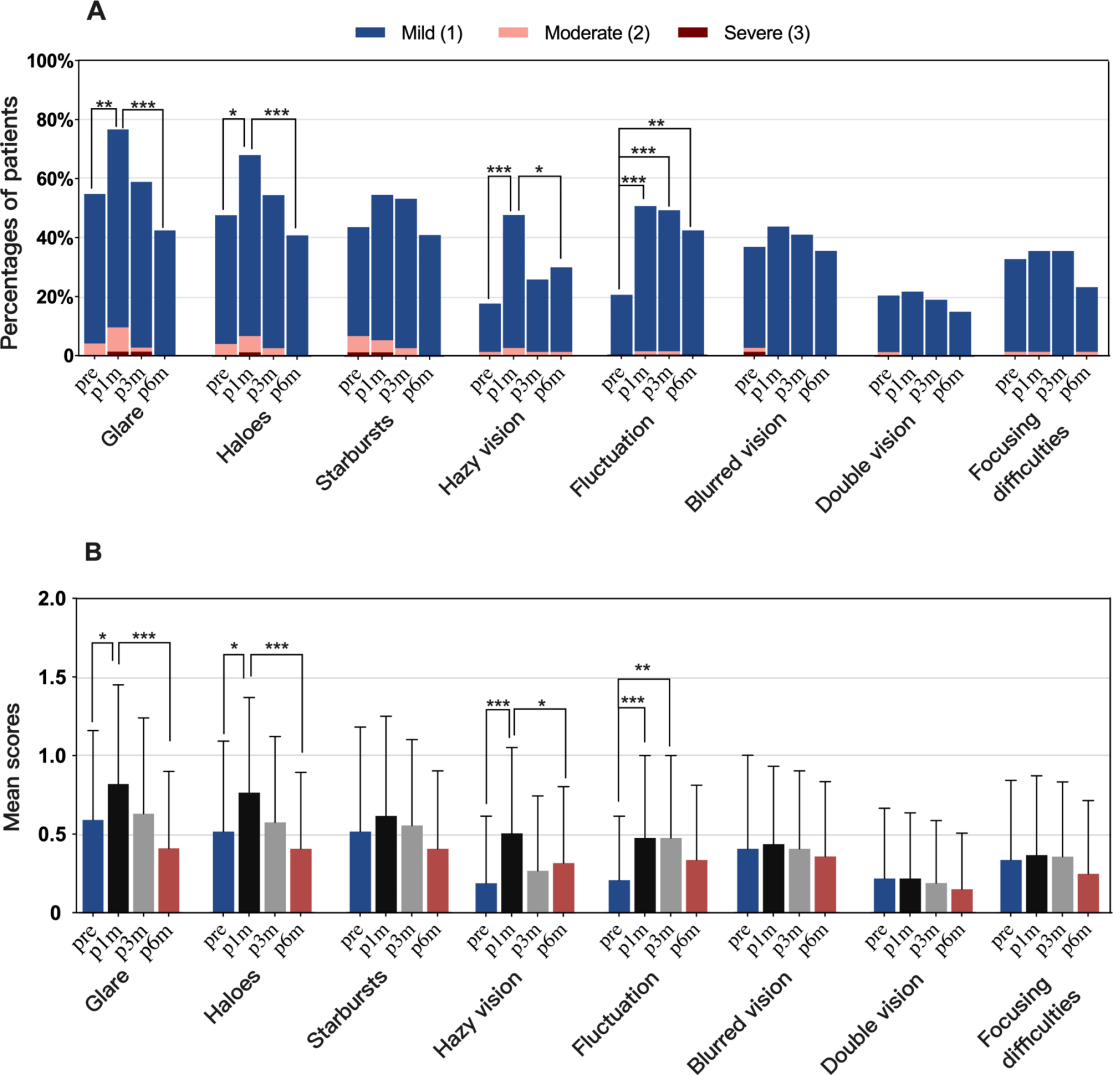
A: The incidence of visual symptoms before and after SMILE; B: The mean extent scores of visual symptoms before and after SMILE. The generalized estimating equation (GEE) with Sidak post hoc test was applied to compare the incidence and extent scores of visual symptoms at different time points; *, ** and *** indicated *P* < 0.05, *P* < 0.01 and *P* < 0.001, respectively.

Figure 3 A showed the incidence of visual symptoms at different time points, and Fig. 3B provided the mean extent scores of symptoms over time.

Before surgery, glare (80 eyes, 55%), haloes (70 eyes, 48%), starbursts (64 eyes, 44%) and blurred vision (54 eyes, 37%) were the four most prevalent symptoms (Fig. 3A). After SMILE, glare, haloes, starbursts and fluctuation were the four most common symptoms from 1 month to 6 months postoperatively (Fig. 3A). And at 6 months, all patients with these four symptoms reported mild discomfort.

For glare, haloes and hazy vision, as shown in Fig. 3A, their incidence rose dramatically 1 month postoperatively compared to preoperative levels (glare: 55% vs. 77%, *P* = 0.002; haloes: 48% vs. 69%, *P* = 0.01; hazy vision: 18% vs. 48%, *P* < 0.001). And at 3 months postoperatively, their incidence returned to baseline (glare: 59%, *P* = 0.99; haloes: 55%, *P* = 0.90; hazy vision: 26%, *P* = 0.63). In addition, compared with 1 month, their incidence decreased significantly at 6 months postoperatively (glare: 43%, *P* < 0.001; haloes: 41%, *P* < 0.001; hazy vision: 30%, *P* = 0.016).

Furthermore, the extent scores of glare, haloes and hazy vision increased significantly at 1 month compared to baseline, as shown in Fig. 3B (glare: 0.59 ± 0.57 vs. 0.82 ± 0.63, *P* = 0.014; haloes: 0.52 ± 0.58 vs. 0.77 ± 0.61, *P* = 0.015; hazy vision: 0.19 ± 0.43 vs. 0.51 ± 0.55, *P* < 0.001). And at 3 months postoperatively, their extent scores returned to preoperative level (glare: 0.63 ± 0.61, *P* = 0.998; haloes: 0.58 ± 0.55, *P* = 0.972; hazy vision: 0.27 ± 0.48, *P* = 0.683). Additionally, compared with 1 month, their extent scores decreased significantly at 6 months postoperatively (glare: 0.41 ± 0.49, *P* < 0.001; haloes: 0.41 ± 0.49, *P* < 0.001; hazy vision: 0.32 ± 0.49, *P* = 0.022).

Concerning fluctuation, its incidence rose significantly 1, 3 and 6 months postoperatively, compared with baseline (Fig. 3A; 21%, 51%, 49% and 43% at baseline, 1, 3 and 6 months; *P* < 0.001 for pre-op vs. 1-month post-op and for pre-op vs. 3-month post-op, and *P* = 0.002 for pre-op vs. 6-month post-op). In addition, its extent scores increased significantly at 1 and 3 months (Fig. 3B; 21 ± 0.41, 0.48 ± 0.53 and 0.48 ± 0.53 at baseline, 1 and 3 months, respectively; *P* < 0.001 for pre-op vs. 1-month post-op, and *P* = 0.001 for pre-op vs. 3-month post-op). And at 6 months postoperatively, its extent scores restored to baseline (0.34 ± 0.48 at 6 months; *P* = 0.195).

The incidence and extent scores of other four symptoms (starbursts, blurred vision, double vision and focusing difficulties) remained unchanged at 1, 3 and 6 months after SMILE, compared with preoperative values (Fig. 3A and B).

### Factors influencing postoperative visual symptoms

The potential influencing factors for postoperative visual symptoms, including preoperative visual symptoms, age, scotopic pupil size, preoperative SE, angle kappa, postoperative ocular HOAs (coma, SA and trefoil), OSI, MTF_cutoff_ and SR were all included in GLMM analysis.

For all analyzed symptoms (glare, haloes, starbursts, hazy vision, blurred vision, double vision, fluctuation and focusing difficulties), the preoperative visual symptoms significantly associated with the extent of postoperative visual symptoms (Table 2). Patients reporting a certain visual symptom preoperatively tended to have higher postoperative scores for the corresponding symptom, compared to patients without that symptom preoperatively. Besides, older patients had higher postoperative scores for double vision than younger patients (Table 2).

**Table 2:** Impacts of preoperative visual symptoms and age on the postoperative visual symptom scores

		With corresponding visual symptom preoperatively	Age
Glare	coefficient (95%CI)	2.02 (0.67, 3.37)	-0.07 (-0.19, 0.04)
	*P*	0.003^*^	0.19
Haloes	coefficient (95%CI)	2.37 (1.06, 3.67)	0.05 (-0.06, 0.16)
	*P*	< 0.001^*^	0.37
Starbursts	coefficient (95%CI)	2.93 (1.60, 4.27)	-0.07 (-0.18, 0.04)
	*P*	< 0.001^*^	0.23
Hazy vision	coefficient (95%CI)	2.89 (1.37, 4.42)	0.06 (-0.04, 0.16)
	*P*	< 0.001^*^	0.22
Blurred vision	coefficient (95%CI)	1.65 (0.41, 2.84)	0.08 (-0.03, 0.19)
	*P*	0.009^*^	0.13
Double vision	coefficient (95%CI)	3.20 (1.71, 4.68)	0.12 (0.002, 0.23)
	*P*	< 0.001^*^	0.046^*^
Fluctuation	coefficient (95%CI)	1.85 (0.36, 3.34)	0.07 (-0.03, 0.17)
	*P*	0.02^*^	0.15
Focusing difficulties	coefficient (95%CI)	2.32 (1.01, 3.63)	0.03 (-0.08, 0.14)
	*P*	0.001^*^	0.56

The generalized linear mixed model (GLMM) was used to explore the influencing factors for postoperative visual symptom scores. The model incorporated various factors, including preoperative visual symptoms, age, scotopic pupil size, preoperative SE, angle kappa, postoperative HOAs, OSI, MTF_cutoff_ and SR. Notably, this table exclusively presents the impacts of preoperative visual symptoms and age. ^*^ indicated *P* < 0.05.

**Fig. 4:**
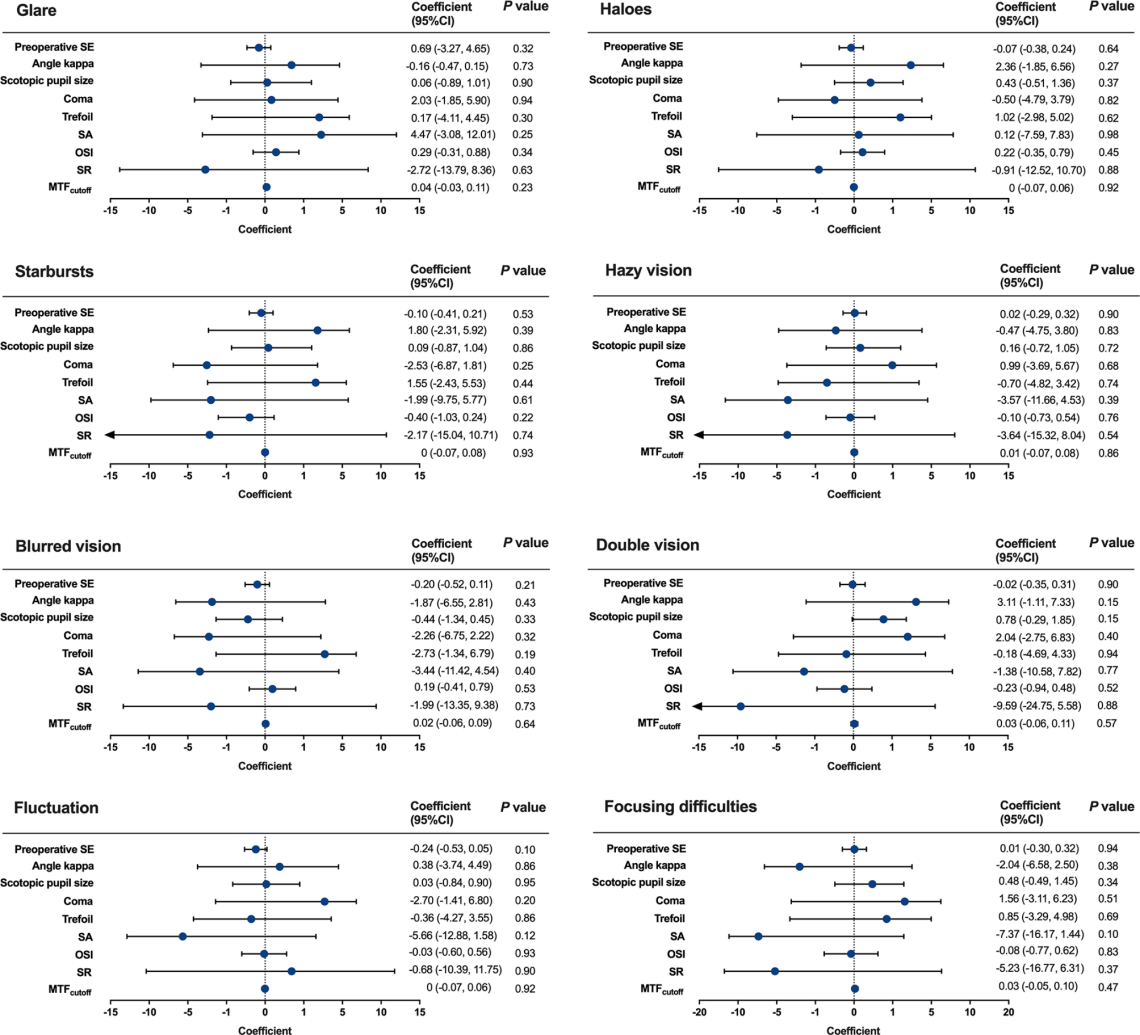
Impacts of preoperative SE, scotopic pupil size, angle kappa, ocular HOAs and scattering indexes on postoperative scores of visual symptoms. The generalized linear mixed model (GLMM) was used to identify the influencing factors for postoperative visual symptom scores. MTF_cutoff_**=** modulation transfer function cut-off frequency; OSI = objective scattering index; SA = spherical aberration; SE = spherical equivalent; SR = Strehl ratio

However, the preoperative SE, angle kappa, scotopic pupil size, postoperative coma, SA, trefoil, OSI, MTF_cutoff_ and SR did not present significant associations with the postoperative scores of above-mentioned visual symptoms (Fig. 4).

## Discussion

Our results showed that glare, fluctuation in vision, starbursts and haloes were the four most prevalent visual symptoms from 1 to 6 months after SMILE. Agreeing with our study, glare was reported as the most common symptom at 3 and 6 months after SMILE [6, 7]. And Schmelter observed that fluctuation (73%) and glare (66%) were the most prevalent complaints after SMILE at a mean follow-up of 24.4 months [5]. However, these studies, like most previous studies, lacked data of visual symptoms before SMILE.

The measurements of baseline visual symptoms are critical, as their absence might lead to overestimation and exaggeration of the actual surgery-induced visual complaints. As a result, it would be unable to accurately assess the real conditions of visual symptoms brought on by SMILE. In our study, preoperative visual symptoms, such as glare, haloes and starbursts were quite common (55%, 48% and 44% respectively). It was also reported that myopic patients corrected with spectacles and contact lenses had visual disturbances [15–17]. Eydelman indicated 73% myopic patients had at least one symptom of double vision, glare, haloes or starbursts [18].

Therefore, this study combined postoperative and preoperative data, to demonstrate the actual dynamic changes of visual symptoms following SMILE. And our results revealed that most of the visual symptoms reported after SMILE also existed preoperatively, and the SMILE-induced visual symptoms were mild and reversible.

As for glare and haloes, their incidence and extent scores rose significantly at 1 month, and returned to baseline at 3 months. It indicated that SMILE-induced glare and haloes were much milder than expected, even though they were the two of the most common postoperative symptoms. Concerning hazy vision, there was a temporary worsening in its incidence and extent scores at 1 month, followed by a recovery to preoperative level at 3 months. The interface healing might explain the dynamic changing pattern of hazy vision in the early post-SMILE period [19]. In terms of fluctuation, its incidence and extent scores worsened significantly at 1 and 3 months. At 6 months, though its incidence was still higher than baseline, the extent scores had restored to preoperative levels. Although SMILE was thought to have a less pronounced impact on ocular surface and corneal innervation [20], the effects of post-SMILE dry eye on vision fluctuation need to be further confirmed [21].

In addition, there were no significant changes in incidence or extent scores of other four symptoms (including starbursts, blurred vision, double vision and focusing difficulties) before and 1, 3, 6 months after SMILE. In the absence of preoperative data, starburst was usually thought to be more pronounced after SMILE. However, as demonstrated by our result, since the preoperative starburst was considerable, the actual starburst did not significantly increase compared to the preoperative one. Once again, the importance of baseline visual symptoms was highlighted. Recently, Reinstein et al. reported that there was a rise in visual complaints at 12 months after SMILE compared to baseline, mainly starbursts and haloes [22]. However, the mean attempted SE of their study population was − 10.55 ± 1.00 D, which was much higher than ours, and might explain the differences. Besides, their study focused solely on visual symptoms at two time points, but neglecting the transitional process within them.

Furthermore, it was worth noting that preoperative visual symptoms were associated with the postoperative extent of all analyzed symptoms. Patients reporting a certain visual symptom preoperatively were predisposed to having a more pronounced corresponding symptom after SMILE, compared to those who had no preoperative visual disturbance. The necessity of preoperative visual symptom evaluation was emphasized by this novel observation. We suggested that preoperative visual symptoms should be fully considered in patient counseling before SMILE.

Besides, our study suggested that the older individuals were more likely to reported more pronounced double vision after SMILE. The effects of age were discussed in Schmeltzer’s study as well, which proposed that patients over 40 years old were prone to more severe postoperative visual disturbances [5]. The effects of age might be attributed to age-related increase in crystalline lens density [23–25], as well as age-related differences in corneal nerve regeneration after SMILE [26, 27]. Further research is needed to better understand the underlying mechanisms behind this association.

For the greater visual result of refractive surgery, it is widely considered that treatment zone should be centered at the visual axis. Due to the lack of an eye tracking system in SMILE, precise centration was required, particularly in situations with apparent angle kappa. Previously, Shao argued that adjustment of angle kappa during the SMILE procedure induced less vertical coma at 5- and 6-mm pupils [28]. And in our study, angle kappa was not associated with postoperative visual symptoms, which might benefit from intraoperative adjustment for eyes with significant angle kappa. This further hinted that angle kappa correction during operation might not only improve the postoperative objective visual quality, but also the patient report outcome.

There were no associations between ocular HOAs and postoperative visual symptoms within 6 months after SMILE in our study. Similarly, postoperative ocular HOAs had no significant associations with haloes, glare, hazy vision, blurred vision and fluctuation at 6 months and 1 year after SMILE [7, 8]. Siedlecki also argued that the associations between postoperative corneal SA and starbursts were weak and clinical negligible [13]. It could be inferred that the interactions between HOAs and visual symptoms were complex, rather than one-to-one correspondences. Another possible explanation might be postoperative neural compensation for the surgery-induced HOAs [29].

The role of pupil size in the visual outcomes of refractive surgery has long been debated. Previous studies showed that a larger pupil size increased aberration, resulting in worse night vision [30]. However, pupil size was not associated with postoperative visual symptoms in our study. Similarly, Schmelter and Li reported there was no correlation between preoperative pupil size and visual quality questionnaire scores after SMILE [5, 11]. The possible reason was that the visual quality questionnaire evaluated patients’ perception in real daily life. And the night time pupil size in real life is smaller than the scotopic pupil size we measured, due to the widespread usage of lighting facilities. Besides, it was suggested that the effective optical zone of SMILE might be significantly larger than LASIK [31]. Thus, the discrepancy between optical zone and pupil diameter may be insufficient to influence visual results after SMILE.

Up to now, the relationship between preoperative SE and visual symptoms still remained controversial. Li reported that preoperative spherical diopters were correlated to postoperative nighttime visual satisfaction [11]. However, our result showed that preoperative SE was not the risk factor for visual symptoms after SMILE. This is probably because when dealing with cases with high myopia, we made every effort to balance cutting depth, optical zone and pupil size, in order to avoid a too small optical zone. Besides, we adjusted kappa angle during operation, greatly reducing off-center cutting, which could be another reason.

There are several options available for correcting myopia and myopic astigmatism. In addition to SMILE, wavefront-optimized and topography-guided laser visual correction (LVC) can be utilized. Wavefront-optimized is aimed at reducing abnormal wavefronts, while topography-guided targets optimal corneal curvatures [32]. Studies have shown that wavefront-optimized can improve low-contrast visual acuity and topography-guided LVC can effectively minimize surgically-induced HOAs [33, 34]. Additionally, topography-guided LASIK has been found to significantly improve glare, starbursts and fluctuation at 12 months postoperatively compared to pre-operation [35]. Therefore, customized LVC is another viable choice for patients with significant preoperative visual symptoms.

The study has some limitations. We did not find significant difference in the extent of some visual symptoms, such as starburst, between preoperative and postoperative 1, 3 and 6 months. However, it is note worthy that there may be a temporary increase in these symptoms at the very early postoperative period (e.g., within the first week after SMILE), which was not evaluated in this study. Another limitation of this study is the lack of evaluation of dry eyes as a factor that may influence visual symptoms. Although the dry eye caused by SMILE is mild [20].

## Conclusions

Most visual symptoms reported after SMILE existed preoperatively, and the lack of preoperative data would overestimate SMILE-induced visual symptoms. Actually, SMILE-induced symptoms were mild and reversible. At 3 or 6 months after SMILE, hazy vision, glare, haloes and fluctuation would return to preoperative level. In addition, preoperative visual symptoms were associated with postoperative visual complaints, patients reporting visual symptoms preoperatively predisposed to more pronounced symptoms after SMILE. Therefore, visual symptoms evaluation ought to be included in the preoperative counseling of SMILE. Furthermore, there were no significant associations between postoperative visual symptoms and preoperative SE, scotopic pupil size, angle kappa (with intraoperative adjustment), postoperative HOAs or scattering indexes.

## Electronic supplementary material

Below is the link to the electronic supplementary material.

Supplementary Material 1

Supplementary Material 2

## Data Availability

The data that support the findings of this study are available on request from the corresponding author upon reasonable request.

## Abbreviations

GEE: Generalized estimating equation
GLMM: Generalized linear mixed model
HOAs: Higher order aberrations
LASIK: Laser in situ keratomileusis
LVC: Laser visual correction
MTF: Modulation transfer function cut-off frequency
OSI: Objective scattering indexes
RMS: Root mean square
SA: Spherical aberration
SE: Spherical equivalent
SMILE: Small incision lenticule extraction
SR: Strehl ratio
