# Supplementary material for: The dynamic changes and influencing factors of visual symptoms after small incision lenticule extraction
Source: BMC Ophthalmol. 2023 May 19;23:223. doi: 10.1186/s12886-023-02964-8 (PMC10197487; doi:10.1186/s12886-023-02964-8)
Supplement: Supplementary file 1 — Supplementary Material 1 [file 12886_2023_2964_MOESM1_ESM.pdf]

Supplemental Table 1 Refractive outcomes after SMILE

Parameters	Mean ± SD			*P* value[a]
	1 month	3 months	6 months	
UDVA (logMAR)	-0.04 ± 0.07	-0.06 ± 0.08	-0.05 ± 0.10	0.85
CDVA (logMAR)	-0.07 ± 0.04	-0.08 ± 0.04	-0.07 ± 0.04	0.91
SE (D)	-0.12 ± 0.45	-0.08 ± 0.43	-0.14 ± 0.38	0.98
Safety index	1.12 ± 0.10	1.13 ± 0.12	1.11 ± 0.10	0.92
Efficacy index	1.05 ± 0.15	1.09 ± 0.17	1.08 ± 0.16	0.69

The generalized estimating equation (GEE) with Sidak post hoc test was used to compare surgery outcomes between postoperative time points; [a] indicated 6 months postoperatively versus 1 month postoperatively. CDVA = corrected distant visual acuity; efficacy index = ratio of postoperative UDVA to preoperative CDVA; logMAR = logarithm of the minimum angle of resolution; safety index = ratio of postoperative CDVA to preoperative CDVA; SE = spherical equivalent; UDVA = uncorrected distant visual acuity.
