# Supplementary material for: The dynamic changes and influencing factors of visual symptoms after small incision lenticule extraction
Source: BMC Ophthalmol. 2023 May 19;23:223. doi: 10.1186/s12886-023-02964-8 (PMC10197487; doi:10.1186/s12886-023-02964-8)
Supplement: Supplementary file 2 — Supplementary Material 2 [file 12886_2023_2964_MOESM2_ESM.pdf]

Appendix: an example of questionnaire

<table>
<tr><td colspan="2">People have different experiences with their vision. Please indicate whether you have following visual symptoms currently.</td></tr>
<tr><td>1. What is the severity of <u>glare</u>?

Right eye ☐No glare ☐Mild ☐Moderate ☐Severe

Left eye ☐No glare ☐Mild ☐Moderate ☐Severe</td><td>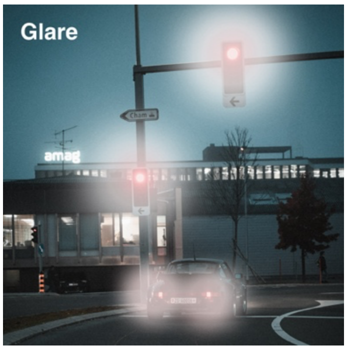
</td></tr>
<tr><td>2. What is the severity of <u>haloes</u>?

Right eye ☐No haloes ☐Mild ☐Moderate ☐Severe

Left eye ☐No haloes ☐Mild ☐Moderate ☐Severe</td><td>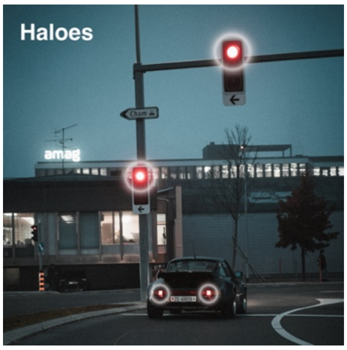
</td></tr>
<tr><td>3. What is the severity of <u>starbursts</u>?

Right eye ☐No starbursts ☐Mild ☐Moderate ☐Severe

Left eye ☐No starbursts ☐Mild ☐Moderate ☐Severe</td><td>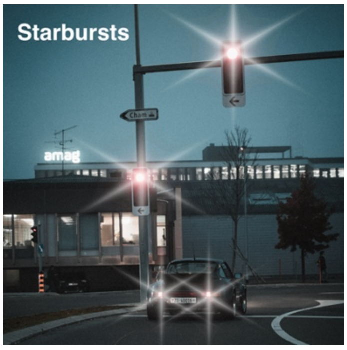
</td></tr>
<tr><td>4. What is the severity of <u>hazy vision</u>?

Right eye ☐No hazy vision ☐Mild ☐Moderate ☐Severe

Left eye ☐No hazy vision ☐Mild ☐Moderate ☐Severe</td><td>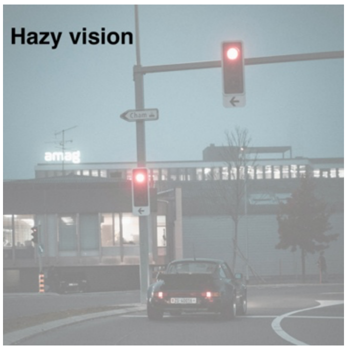
</td></tr>
</table>

5. What is the severity of **<u>blurred vision</u>**?

Right eye ☐No blurred vision ☐Mild ☐Moderate ☐Severe

Left eye ☐No blurred vision ☐Mild ☐Moderate ☐Severe

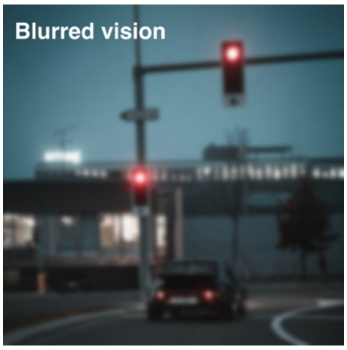

6. What is the severity of **<u>double vision</u>**?

Right eye ☐No double vision ☐Mild ☐Moderate ☐Severe

Left eye ☐No double vision ☐Mild ☐Moderate ☐Severe

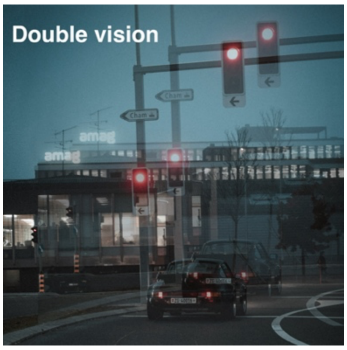

7. What is the severity of **<u>fluctuation in vision</u>**?

Right eye ☐No fluctuation in vision ☐Mild ☐Moderate ☐Severe

Left eye ☐No fluctuation in vision ☐Mild ☐Moderate ☐Severe

8. What is the severity of **<u>focusing difficulties</u>**?

Right eye ☐No focusing difficulties ☐Mild ☐Moderate ☐Severe

Left eye ☐No focusing difficulties ☐Mild ☐Moderate ☐Severe
